# Water-related limits to growth for agriculture in Iran

**DOI:** 10.1016/j.heliyon.2023.e16132

**Published:** 2023-05-17

**Authors:** Mostafa Khorsandi, Tayebeh Omidi, Pieter van Oel

**Affiliations:** a Centre Eau Terre Environnement, Institut National de La Recherche Scientifique, 490 rue de la Couronne, Québec, G1K 9A9, Canada; b Canadian Rivers Institute, UNB Fredericton, 28 Dineen Dr Fredericton, New Brunswick, E3B 5A3, Canada; c Water Resources Management Group, Wageningen University, Wageningen, PO Box 47 6700AA, Netherlands; d Water Resources Engineering Group, Tarbiat Modares University, Tehran, Iran

**Keywords:** Water footprint, Virtual water, Agriculture, Satellite imagery, Iran

## Abstract

Globally, agriculture is the primary water consumption sector. This study used water footprint (WF) as a bottom-up tool and satellite imagery as a top-down tool to estimate the internal water use (WU) in the agricultural sector in an innovative way to show the effects of water-intensive use in agriculture in an arid country. The WF of Iran has been quantified for 19 main crops and for related agricultural products exported from Iran to partner countries. Using a bottom-up approach, Iran's total yearly agriculture net water consumption is estimated to be 42.43 billion cubic meters (BCM) per year. Out of 42.43 BCM total net internal water use, only 1.61 BCM is virtual-water export related to these 19 products, and the remaining 40.82 BCM is for internal use. Our results using satellite imagery show that in case of using all possible lands for agriculture, it would require 77.4 BCM. However, not all these lands are within human reach, and the maximum available water is way lower than this amount. Using satellite imagery, the total evaporation from agricultural lands shows 55.27 BCM for 2020, which agrees with national reports during 2005–2014. This study shows that agricultural water consumption tends to use internal water resources at a maximum level for export and national use, significantly impacting renewable and non-renewable water resource availability, especially in groundwater.

## Highlights

• Water footprint was used to estimate water use for agriculture for main crops.
• An Earth observation method was used to assess water use for all croplands.
• A land versus evapotranspiration method was developed to show water bankruptcy.
• Iran suffers a collapse in groundwater resources due to agricultural policy.
• The equilibrium state of the agriculture system was shown using the developed method.

## Graphical abstract

**Figure:**
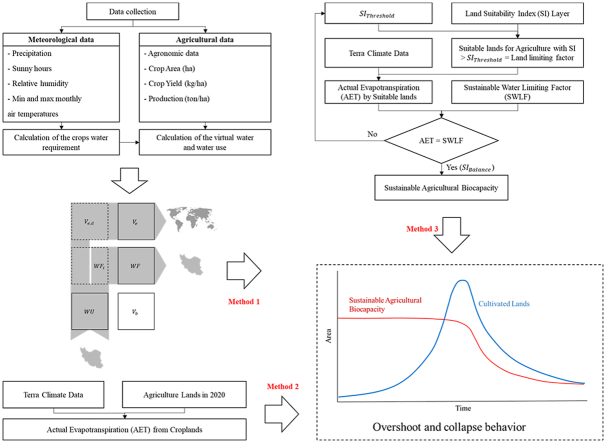

## 1 Introduction

Agriculture is the basis of food security worldwide [1,2]. With an increasing population and finite resources, the extent of agricultural growth is vital for sustainability while respecting natural limits [3,4]. Water, soil, and photosynthesis are the main limiting factors in agricultural production [5]. Among these factors, water acts as the bottleneck for arid countries [6]. In these countries, using domestic water resources for producing crops for internal consumption and export is a major contributor to water-related problems [7].

Currently, about 9% of Iran's land is devoted to agricultural production, which is responsible for 55.27 billion cubic meters (BCM) net water consumption as evapotranspiration [5]. At the same time, water availability in Iran has decreased due to climate change impacts (e.g., reduced precipitation and increased temperature and evaporation) [[8], [9], [10], [11]]. However, despite decreased water availability, the irrigated agricultural lands have maintained or increased during the last decades [8]. This unsustainable agricultural development has been possible by the over-abstraction of surface and groundwater resources [8]. Consequently, unsustainable agricultural land development at this level in Iran as an arid/semi-arid country caused two main challenges, including drying rivers and lakes [[12], [13], [14]] and severe groundwater (GW) drawdown [8,[15], [16], [17], [18], [19]]. Investigations of river flow changes in Iran over time and space showed that about 56% of stations have a decreasing trend in river flow (2.5 times the global average) [20].

Previous studies showed despite decreasing water availability, agricultural production in Iran has increased over the 1981–2013 period [8]. This expansion was facilitated by the excessive use of non-renewable water resources, which has a significant environmental impact on water quantity [20,21] and quality [22]. Moreover, water bankruptcy in Iran has severe socioeconomic consequences (e.g., high unemployment rates, dependence on oil and gas exports, and damages to the infrastructures) [23,24]. Therefore, urgent policy reforms are needed to adapt the agriculture sector to natural water availability, ecological carrying capacity, and the expected impacts of climate change in different parts of the country [23,25].

Different countries implemented variations of adaptation or mitigation methods to tackle water-related limits for food production in the agriculture sector. For example, Panyasing et al. [26] results show that implementing the national and provincial level policies has generally advanced agricultural output in the Yasothon province in China. However, their main focus was on a single crop. Also, Abidin et al. [27] investigated the effects of agricultural irrigated areas, import of raw materials, labor, and capital development on crop production, focusing on rice production in the Malaysian agricultural system. According to their results, high production can be improved by using irrigated agricultural lands and importing raw materials, labor, and capital. These results can be generalized to the conditions of Iran for the production of major products because these challenges have resulted from intensified management strategies, such as agricultural development plans, subsidizing energy, self-sufficiency voice, using agriculture as a labor force market, and improving cropping systems’ productivity [28].

One of the policies to tackle water-related problems in agriculture is to use new technologies. Due to climate change and the growing population, determining the appropriate strategy and technology for irrigation is necessary. Dwijendra et al. [29] showed that in the non-stress irrigation method, crop production and net profit are almost equal in traditional and modern irrigation methods. However, under water stress conditions on the plants, modern irrigation technologies increased crop production and net profit 1.75 times more than traditional methods, indicating irrigation technology's impact on crop production. However, although irrigation efficiency saves water at the farm scale, this surplus water usually causes a rebound effect, which means more investment in cropland developments. Therefore, the rebound without following ecological limits causes an increase in the irrigated area [30,31]. The increased irrigated area at the basin scale increases the effective evaporation from croplands. Therefore, the only solution at the large basin scale is to cap the croplands area while improving the irrigation efficiency via irrigation technologies [30]. Knowing these facts, Mesgaran et al. [25] proposed a national adaptation plan to tackle water scarcity in Iran. They showed increasing water withdrawals over the last three decades which overshoot the availability of water resources. By offering the land suitability for agriculture and the water stress level in sub-basins, they mentioned the commodity import (virtual water import for Iran) necessary for an effective water scarcity adaptation. However, Iran's current situation shows a self-sufficiency policy in agriculture [5].

The state of food security, achieved under intensified strategies, cannot last long. With a possible collapse in production, the agricultural system will finally reach a new lower and collapsed equilibrium state [5]. Analyzing the collapse situation needs a holistic approach focusing on all agricultural production cycles. Water footprint (WF) is a multidimensional indicators assessment method related to water consumption [[32], [33], [34]], which can be used to show consumption trajectories related to growth in agriculture using multiple agricultural products [35,36]. There are various studies related to WF assessment in Iran. Mirzaie-Nodoushan et al. [37] conducted a study on the impact of diet on the national water footprint of Iran. The study revealed that a change in diet resulted in a decrease of 4.5–7.8 BCM per year in water consumption. However, this reduction is relatively insignificant compared to the decreased surface and groundwater resource volume.

Similarly, Karandish et al. [28] analyzed two primary scenarios for virtual water trade in Iran. Their findings based on WF assessment for 27 major crops showed that 8.45–14.41 BCM per year of exported water footprint comes from blue water resources resulting from water mismanagement. Previous studies have used WF assessment for Iran, but none considered blue and green water resources for agricultural production simultaneously at the national scale. In addition to the WF assessment, recent studies have proven that satellite imagery is an efficient method for large/national-scale analysis of water use in croplands [5,38].

The evidence indicates a lack of a common narrative regarding the limits to agricultural sector growth in Iran. Most of Iran's available water is committed to high agricultural development ambitions aiming for self-sufficiency without adequately considering natural limitations [13,14]. Not only is domestic crop production not enough for internal use, but Iran is also a leading exporter of virtual GW through the export of agricultural products [7], leading to decreasing water availability [10,19,39]. The impacts vary from drying rivers and lakes, declining lake levels and GW tables, and increased saltwater intrusion in coastal areas [13].

As a bottom-up approach, WF can be merged with top-down satellite imagery analysis for a more holistic understanding of the agricultural system growth limits. To our knowledge, no previous study has combined these two approaches to show the limits to growth for the agricultural sector in Iran. This study aims to show the limits of agricultural production due to water availability in Iran as a well-studied arid/semi-arid country confronted with policy challenges around food and water. This study aims to address this research gap by revealing the impact of water availability on the limits of agricultural production in Iran.

## 2 Materials and methods

As a tool to show the water use related to agriculture, we use the WF concept [32]. The agricultural WF of a nation is defined as “*the total amount of freshwater that is used to produce crops consumed by the inhabitants of the nation*” [33]. The total WF of a country has two parts: (1) the internal WF coming from local resources and (2) the external WF from other countries via crop imports [33,37]. In this study, we focus on the internal WF of Iran.

The internal WF of Iran for agriculture is the amount of water used to produce crops consumed by Iran's inhabitants or exported to other trade partner countries. The total internal WF is a quantitative tool to show the amount of water consumed locally or exported via international trade. The consumptive water use can consist of use from both blue and green water resources, corresponding to the blue and green WF s respectively. The blue internal WF of agriculture is the amount of freshwater that evaporates from the local blue water resources [surface water (SW) and groundwater (GW)] to produce crops. The green internal WF is the amount of water evaporated from the local green water resources (mainly effective rainwater stored in the soil as soil moisture) [40].

The analysis period of the current study is 2005–2014, which overlaps with global reanalysis data. The average of this period can provide good insight into the core reasons for the looming water crisis in Iran [14] and the roots of the current environmental collapse [5]. At the same time, this period is long enough to provide a trajectory of Iran's policy around agricultural production.

### 2.1 Method implementation

**Fig. 1:**
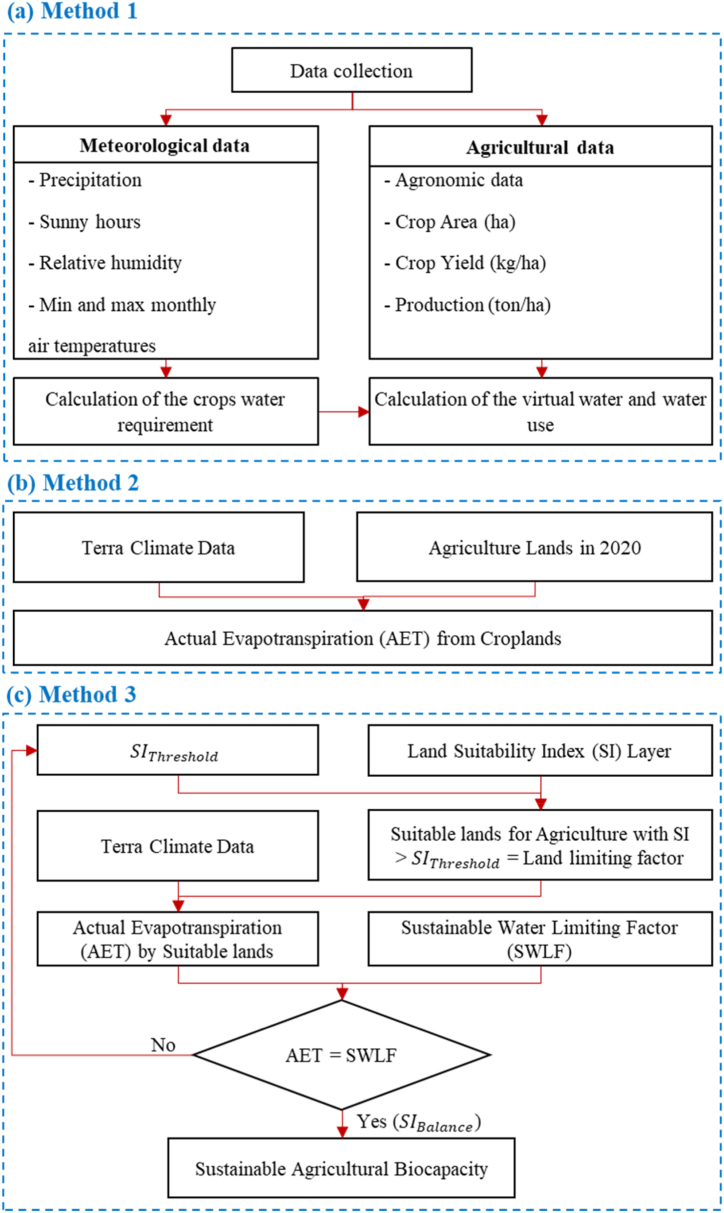
Overall methodological flowchart of this study including (a) the bottom-up approach using water footprint assessments (Method 1), (b) the first top-down method using satellite observations on croplands in 2020 (Method 2), and (c) the second top-down method using suitable lands for agriculture (Method 3).

We assessed Iran's agricultural growth limits using a method among bottom-up approaches (water footprint accounting) and two methods among top-down approaches (using satellite imagery and global scale databases). In the first method, by using 19 main agricultural products in Iran, we estimated domestic water use for agriculture, internal water footprint, and the exported water footprint from internal water resources. For the second method, we first produced agricultural land suitability for the whole country using available global geographic information system databases and the Google Earth Engine platform. The Iran Ministry of Energy provides the water limit at the basin scales, which calculates the maximum amount of water available for consumption at different spatial levels. The first top-down method shows the possible agricultural lands as a function of the suitability index (SI) value. This method shows the trajectory of agricultural water use based on agricultural land areas. The second top-down method uses ESRI cropland data for 2020 to estimate the actual evapotranspiration of these lands. Finally, we discuss that agricultural lands reported by the Ministry of Agriculture in Iran are reliable and follow the same result as method 2. Since method 2 is one snapshot of the trajectory in method 3, method three can be used to show the water bankruptcy situation in Iran, overshoot and collapse trajectory, and possible equilibrium states after the collapse for sustainable or unsustainable future scenarios. This overall flowchart is presented in Fig. 1.

### 2.2 Case study

**Fig. 2:**
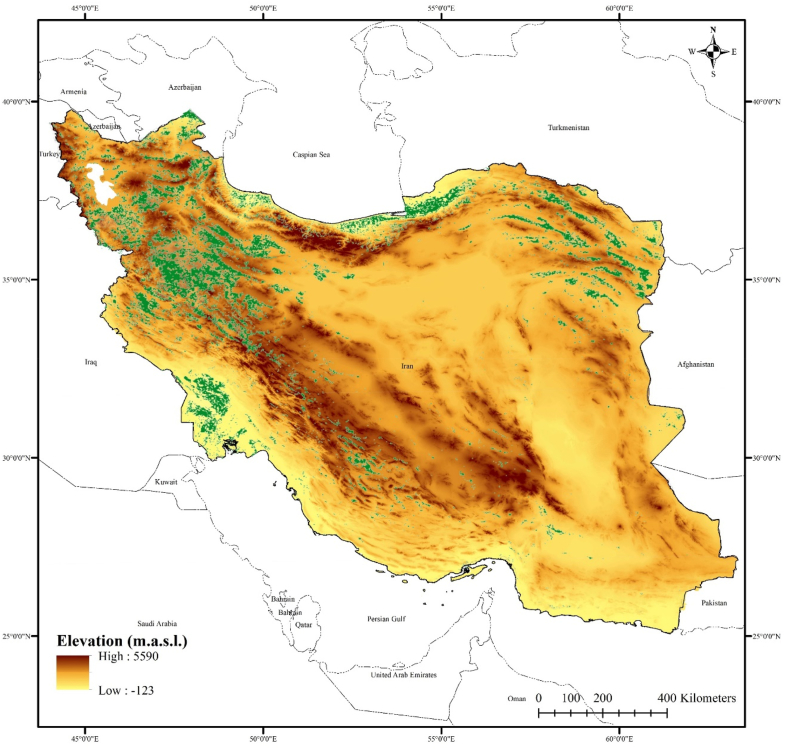
Iran's geographical location, its neighboring countries, and its croplands (in green) for 2020. (For interpretation of the references to colour in this figure legend, the reader is referred to the Web version of this article.)

Iran is a large country in the Middle East with a land area of 1,648,195 km^2^ and a population of 84 million people. Its borders include Iraq, Turkey, Armenia, Azerbaijan, Turkmenistan, the Caspian Sea, the Persian Gulf, the Oman Sea, Afghanistan, and Pakistan (Fig. 2). The country has an arid and semi-arid climate, with an average annual precipitation of 250 mm and 30% of precipitation occurring as snow. Iran has limited surface water and groundwater resources and high geographical heterogeneity in supply and demand (see Fig. 2).

The agricultural sector is the largest water user (green regions in Fig. 2), followed by urban and rural water supply and mining sectors. Iran is currently facing social, economic, and environmental challenges, including a transition from ideologically-driven autocracy towards a more democratic future, high unemployment rates, international sanctions, dependence on oil and gas exports, loss of glaciers due to climate change, excessive groundwater drawdown from agricultural use, and decreased precipitation over the past few decades. These issues have created a complex and wicked problem that affects not only Iranian citizens but also has international implications, particularly for the stability of the Middle East region.

### 2.3 Internal water footprint

**Fig. 3:**
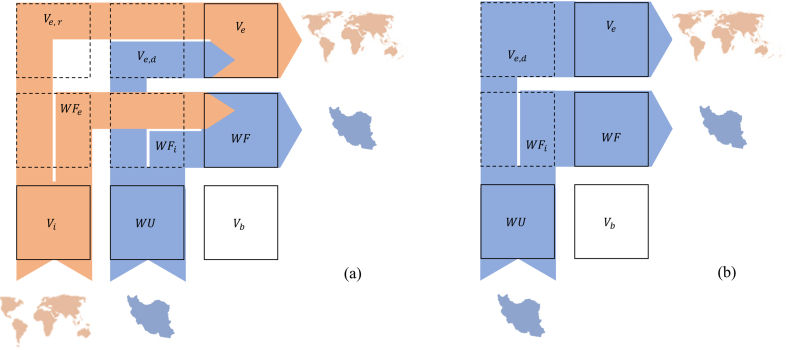
(a) The schematic relationship between imported water footprint ($V_{i}$), internal water footprint (WU), national budget for a water footprint ($V_{b}$) of a country. National internal water use (WU) can be divided into two parts: Internal water footprint (${WF}_{i}$) and virtual water export originated from internal water use ($V_{e,d}$). Typically, an economy re-exports part of $V_{i}$ as $V_{e,r}$ or part of its WU as $V_{e,d}$, (b) The schematic relationship between water footprint and virtual water export for a country by focusing on internal water consumption.

The total water volume from the internal water resources in the national agriculture sector shaped the primary water use (WU). The agricultural WF is the amount of WU for national uses per year for this sector. For crops, WF has the unit of $\frac{m^{3}}{Year}$. For each country, the internal WF budget ($V_{b}$ which is equal to WU by ignoring the imported commodities) has two internal (${WF}_{i}$) and external parts ($V_{e,d}$) [37] as Equation (1) and Fig. 3a:

(1) $$WU=V_{b}={WF}_{i}+V_{e,d}$$

The focus of this research is the total water volume used from the internal water resources in the national agricultural sector (WU), using bottom-up and top-down approaches (see Fig. 3b). Van Oel et al. [33] provided the analytical framework to calculate WU. The WU in the agriculture sector is defined as the annual use of internal water resources to produce farm crops via agriculture.

### 2.4 Bottom-up approach for water footprint calculation

In the bottom-up approach, the WU of the Iran's agriculture (IR) is calculated (Method 1) by summing the direct water use by crops as follows. This step is done without considering their indirect water use (e.g., water used for after-harvest processing, food processing, packaging, and transportation) due to a lack of data according to Equation (2).

(2) $${WU}_{agriculture}\left[IR,n\right]=\sum_{i=1}^{n}\left[P_{i}\times {vwc}_{i}\right]$$

where $P_{i}$ is the production of crop i (unit/year), and ${vwc}_{i}$ is the virtual water content of this crop (m^3^/unit). In the case of geographically explicit production of the same crop, the result is based on Equation (3):

(3) $${WU}_{agriculture,t}\left[IR,n\right]=\sum_{i=1}^{n}\sum_{j=1}^{m}\left[P_{i,j,t}\times {vwc}_{i,j,t}\right]$$

where $P_{i,j,t}$ is the production of crop i in j region in the year t, and ${vwc}_{i,j,t}$ is the virtual water content to produce the same product in the region on the same year. The accuracy of this method depends on the completeness of the databases for all crops. In this study, 19 main crops were considered. Justification for selecting these 19 crops is provided in “Crop selection” section.

The virtual-water content of a crop product is the amount of freshwater (Blue and Green water) evaporated and transpired to produce the crop, measured at the place where the product was produced (provinces). The virtual-water content of a crop varies as a function of place and growing season. Therefore, it refers to the sum of the evapotranspiration at different crop growth stages. The adjective ‘virtual’ means most water used to cultivate a crop is not contained in the final harvest. The amount of water content of the harvest is negligible compared to the virtual-water content of the growing season [33].

#### 2.4.1 Crop selection

According to Iran's export and production statistics (Ministry of Agriculture Jihad), the crop products with the highest export amount were considered. The main category of products, names, and the average annual amount of exports are present in Table 1 (19 products).

**Table 1:** The Average annual amount of export from Iran for the main agricultural products.

Category	Crops in each category (values in metric tons)			
Cereals	Wheat	Barley	Rice	Grain corn
	67,894	2	2052	1130
Legumes	Lentils			
	37			
Industrial products	Sugar beet	Soy	Sugarcane	
	8	2010	33,614	
Vegetables	Potatoes		Tomatoes	
	279,607		185,739	
Melons	Watermelon		Melon	
	339,281		42,825	
Fodder plants	Alfalfa	Clover	Fodder corn	
	11,062	69	67	
High-value fruits	Pistachios	Saffron	Dates	Oranges
	234,532	56	73,969	2061

#### 2.4.2 Crop water requirement calculation for the bottom-up approach

Reference crop evapotranspiration (ET0) is a function of location and weather, considering unlimited available water. Based on ET0, the actual crop evapotranspiration (${ET}_{crop}=vwc$) can be calculated as follows based on crop growth stages [Equation (4)]:

(4) $${{vwc}_{i,j,t}=ET}_{i,j,t}=\sum_{s=1}^{S}\left[K_{i,j,t,s}\times {ET0}_{j,t}\right]$$

where $K_{i,j,t,s}$ is the coefficient for crop i for province j, in the year t at the growth stage s; S is the total growth stages for the crop. A national agricultural database was used for $K_{i,j,t,s}$ for all provinces. The water limit imposed by meteorological data at the final step varies spatiotemporally. If effective rain (or irrigation) is higher than $\left[K_{i,j,t,s}\times {ET0}_{j,t}\right]$, the crop would not face any water deficit. Otherwise, the available water would control the real ${ET}_{crop}$. The above steps were done using CROPWAT software for all 19 crops for all 32 provinces using data for a period of ten years (Fig. 1a). For this calculation, the ET from efficient precipitation and ET via irrigation is calculated separately by CROPWAT. This splitting can provide separate values for the green and blue WF s.

#### 2.4.3 Exported virtual water calculation

Similar to crop production, $V_{e,d}=V_{e}$ was calculated. Using the method by Hoekstra and Hung [41], by knowing trading partners and exported amount of crop, $V_{e}$ can be calculated as Equation (5):

(5) $$V_{e,t}\left[IR,n\right]=\sum_{i=1}^{n}\sum_{e=1}^{E}\left[X_{i,e,t}\times {\bar{vwc}}_{i,t}\right]$$

where $X_{i,e,t}$ is the amount of exported crop i in the year t to the traded partner country e (Ton), and ${\bar{vwc}}_{i,t}$ is the virtual water content of crop i across Iran in the year t ($\frac{m^{3}}{ton}$). ${\bar{vwc}}_{i,t}$ is the average value of cultivating provinces for that year, as a representative for the whole country.

### 2.5 Top-down approach

The second approach for assessing the agricultural water footprint of a country (WF, $\frac{m^{3}}{year}$) is the top-down approach, which takes the total water use in the country in agriculture (WU) as starting point and then adds the incoming virtual-water flow ($V_{i}$) and subtracts the virtual-water export ($V_{e}$). For this approach, Method 2 uses satellite imagery for agricultural land delineation and reanalysis data for actual evapotranspiration (Fig. 1b). Method 3 in this approach is to find possible lands for agriculture using the land agricultural suitability index (SI). Then this possible land area multiplied by actual evapotranspiration (AET) from reanalysis data results in AET for agriculture (Fig. 1c).

#### 2.5.1 Crop water requirements for the top-down approach

The TerraClimate reanalysis database (Abatzoglou et al., 2018) was used with monthly time steps to calculate AET for Methods 2 and 3.

#### 2.5.2 The top-down approach using satellite observations (method 2)

ESA World land cover [42] with a 10 m resolution for 2020 was used to delineate the croplands in Iran. By using crop cells area from ESA World land cover and actual evapotranspiration from the TerraClimate reanalysis database, the AET for the year 2020 was calculated according to Equation (6):

(6) $${AET}_{2020}=\sum_{i=1}^{K}A_{i}\sum_{j=1}^{12}{AET}_{i,j}$$

where $A_{i}$ is the area for cell i, and ${AET}_{i,j}$ is actual evapotranspiration for cell i during the month j for 2020.

#### 2.5.3 The top-down approach using agricultural suitability analysis (method 3)

Since ${AET}_{2020}$ is one annual snapshot to estimate the national agricultural water consumption, a second method was used. In the second method, we used the Suitability Index map for agriculture produced by Khorsandi et al. [5] based on Mesgaran et al.'s [43] methodology. In this method, national AET is a function of SI according to Equation (7):

(7) $$\bar{AET}\left(x\right)=\sum_{i=1}^{K}A_{i}\sum_{j=1}^{12}{\bar{AET}}_{i,j},SI\left(A_{i}\right)>x$$

where for each x threshold, $A_{i}$ are cells with the SI value greater than x and ${\bar{AET}}_{i,j}$ is long-term average actual evapotranspiration for cell i during the month j. This function shapes a continuous curve, showing the spectrum of possible $\bar{AET}$ in Iran at the national level which ${AET}_{2020}$ represents one sample of this average.

## 3 Results

### 3.1 The water footprint of Iran's consumers from main crops

Table 1 shows the total water use for agriculture to produce 19 main crops at the national level. The main water use is shaped by blue water (SW and GW). Our results show Iran uses 42.43 BCM (varied from 30.47 to 49.91 BCM during 2005–2014) of internal water resources to produce the main 19 crops. These results show an increasing trend in water use both for national internal consumption and export.

### 3.2 The virtual water export share of Iran's major crop production

**Fig. 4:**
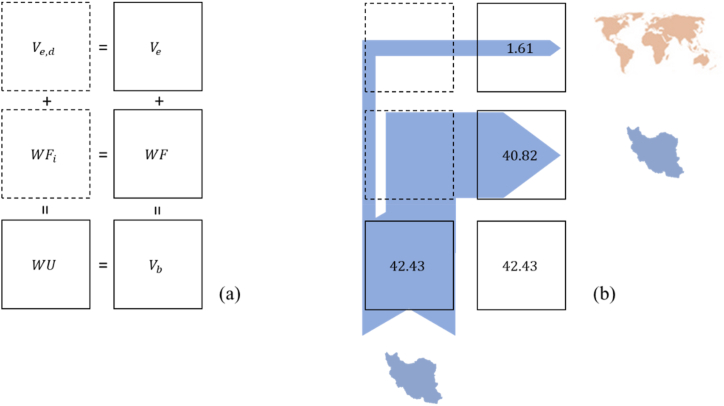
(a) The relations between virtual-water exports ($V_{e}$), use of national water resources (WU) and the water footprint (WF) of Iran. (b) The resulted WF assessment for Iran based on internal water resources. The numbers in the boxes are annual average values of the results for Iran for the period 2005–2014.

Table 2 shows Iran exports on average 1.47 BCM (varied from 0.19 to 3.74 BCM during 2005–2014) of internal water resources as the virtual water content of the major 19 crops. In addition, Table 2 values for exported blue water shows that the exported water mainly has blue water origins (Mainly from irrigated lands, using surface/groundwater resources) (see Fig. 4).

**Table 2:** Iran's nationwide water use, water footprint, and virtual water export for 19 main crops in 2005–2014, and their share from blue and green water [units in billion cubic meters (BCM)].

Blue and Green water footprint					Blue water footprint		
year	WF	$V_{e}$	WU		WF	$V_{e}$	WU
2005	35.60	0.45	36.05		21.82	0.25	22.06
2006	39.12	0.19	39.31		24.05	0.19	24.24
2007	29.54	0.93	30.47		19.13	0.92	20.05
2008	44.92	1.51	46.44		36.53	1.51	38.04
2009	39.41	1.43	40.84		26.80	1.43	28.23
2010	46.17	3.74	49.91		35.87	2.67	38.54
2011	45.89	1.85	47.75		33.74	1.83	35.57
2012	42.21	1.85	44.06		29.42	1.84	31.26
2013	43.09	1.70	44.79		32.83	1.70	34.53
2014	42.28	2.45	44.72		31.39	2.39	33.78
Average	40.82	1.61	42.43		29.16	1.47	30.63

### 3.3 Internal water resources used for agriculture during 2020

**Fig. 5:**
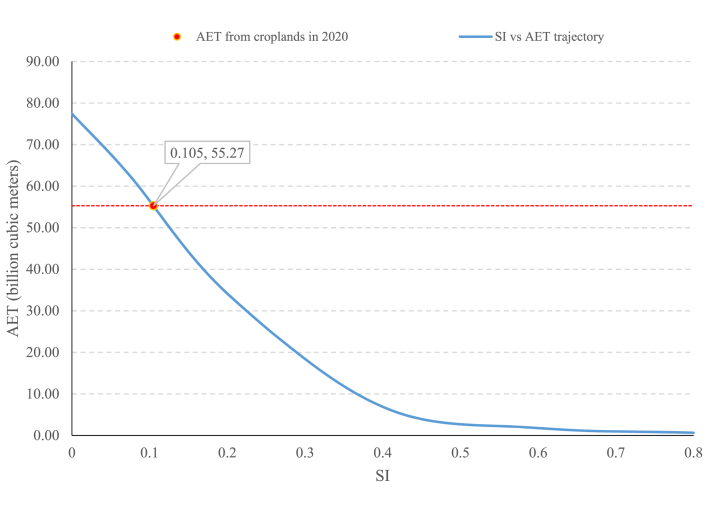
Calculated AET value for 2020 in billion cubic meters and its trajectory based on the quality of cultivated lands using long-term actual evapotranspiration reanalysis data. SI is the suitability index of the lands for agriculture.

The total water use of Iran's croplands for 2020 is about 55.27 BCM. Based on ESA satellite data for Iran, with a 10 m resolution, croplands water use is responsible for the most significant part of Iran's internal water consumption (Fig. 5).

### 3.4 Internal water resources use trajectory in Iran for the agriculture sector

The water use due to agriculture is variable. Not only is there no yearly database for croplands area, but the total crop production is also rarely available. Therefore, one implicit way to estimate possible water use at the national level is to use possible lands. Fig. 5 shows the AET value from possible croplands based on the land suitability index. Fig. 5 shows that in the most extreme way of land allocation for agriculture, Iran's water use in this sector cannot be beyond 77.42 BCM. This extreme mode with SI>0 values mean doing agriculture practice in all the possible lands in Iran, excluding deserts, mountains, and forests. Fig. 5 puts in perspective the limited available lands for agriculture with high suitability index.

## 4 Discussion

The relationship of AET with croplands can be analyzed based on designed scenarios using the methodology proposed by Khorsandi et al. [5]. For this goal, the current AET from croplands for 2020 is plotted, together with thresholds for available national and local water volume in Iran [5]. Based on the ministry of energy (MoE) analysis in Iran, the total national available renewable water equals 61.62 BCM if environmental flows for SW and GW are allocated at the national level. At the same time, by allocating environmental flows at the local level (609 hydrological study units in Iran), this value equals 32.8 BCM.

**Fig. 6:**
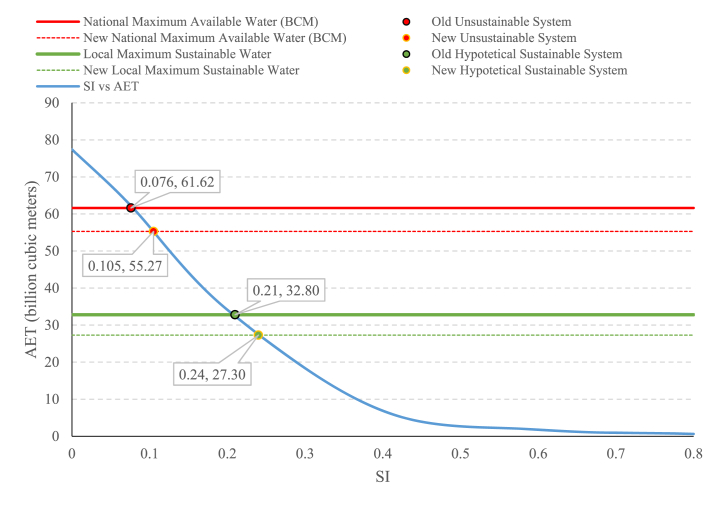
Calculated SI threshold values using a developed trajectory tool and different levels of water availability (in billion cubic meters) based on the Iran's Ministry of Energy reports.

**Fig. 7:**
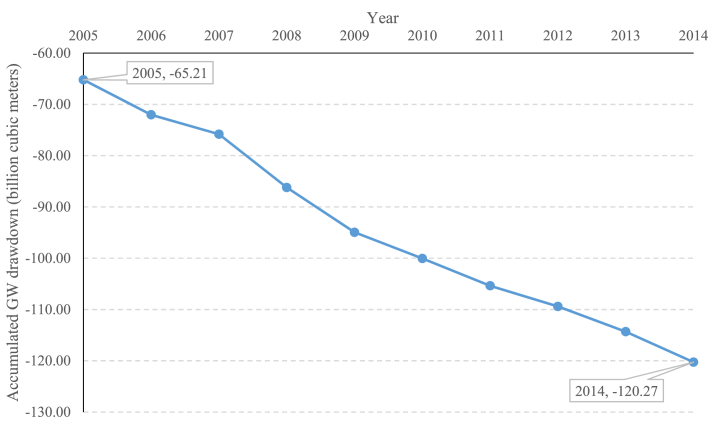
Accumulated groundwater drawdown (in billion cubic meters) at the national level [sum of 609 hydrological response units] during 2005–2014 (reference: Iran's Ministry of Energy).

Fig. 6 shows that theoretically, at the national level, if the national total available water (61.62 BCM) is used to produce agriculture, the national-level environmental flow is theoretically met, and we should, therefore, not expect to see a drawdown in neither GW nor SW levels. However, it is a wrong assumption because water availability and environmental flow (for SW and GW) vary geographically over the country and should therefore be considered locally. Therefore, MoE managed the national water system in an unsustainable manner [“Old Unsustainable System in Fig. 6]. If we consider local water availability situations, the sustainable available water (Sum of 609 study regions) substantially reduces to only 32.8 BCM [5] [Labeled as “Old Hypothetical Sustainable System” in Fig. 6]. Assuming the maximum available water as 61.62 instead of 32.8 cause an unsustainable system with a transition to its equilibrium state. This transition due to unsustainable allocation of water resources can have two effects: (1) drying of local SW bodies (rivers, lakes, and swamps), (2) Local GW drawdowns, with the rate of −5.51 BCM/year at the national scale for 2005–2014 (Fig. 7). Both effects are visible at this moment in Iran. Not only are major SW bodies dried across the country, but official reports on GW drawdown are visible in Fig. 7 [10,16].

At the same time, the land cover for 2020 and the resulting AET using it shows the value of 55.27 BCM [labeled as “New Unsustainable System in Fig. 6], which is lower than 61.62. These results are contradictory and show at least one of the assumptions within national water decision-making is wrong since, during the 2004–2015 period, Iran faced drying SW (rivers and lakes) and GW during this time drawdown of 55.06 BCM, with firm observations (Fig. 7). The assumptions by Iranian decision-makers are as follows.

1 The maximum available renewable water for all uses is 140 BCM, of which 90 BCM is manageable.
2 The total amount of available renewable water for all uses is 61.62 BCM, which excludes environmental flows for SW and GW at the national level.
3 The total amount of available renewable water for all uses, considering local water availability, is 32.8 BCM, excluding environmental flows for SW and GW at the local level at 609 hydrological study areas.
4 GW drawdown does not cause storage loss in the short-term/mid-term due to land subsidence.

Our results show that some of these assumptions need to be revised for the following reasons:

1 The maximum available renewable water for all uses is not 140 BCM and possibly has shrunk. This reduction is due to climatic changes [12,39,44], which take away the available water in at least two forms: (a) precipitation reduction and (b) increased evaporation, which makes less blue and green water accessible [10,45,46]. This possible reduction and climatic regime shift in available water were already shown by Saemian et al. [10].
2 The total amount of available renewable water for all uses is not 61.62 BCM. We argue that water is a local resource and considering 61.62 BCM is therefore not a valid assumption. Our other reasoning is since energy for agriculture is subsidized, water for agriculture is cheap, and there is a voice of self-sufficiency in agriculture, the agriculture sector used all the potential to produce food. This combination of incentives means all possible lands are cultivated, and the possible, manageable amount of green and blue water is converted to evapotranspiration. The environmental flows are at their minimum level or zero. However, with all of this intensive use, the limits to growth of AET show 55.27 BCM for 2020, which is less than 61.62 BCM, and just during the 2005–2014 period caused 55.06 BCM accumulated loss in GW storage.
3 Considering local water availability, the total amount of renewable water for all uses is not 32.80 BCM anymore. This value is based on the long-term water cycle and using the data from 50 years. This value does not consider climate change impacts on water availability and does not consider the accumulated loss in surface and GW losses, which should be recovered this time with less available water. We estimate that if we consider the linear relationship for GW drawdown, the loss rate is −5.51 BCM/year. Therefore, for a yearly water budget, this value should be subtracted from the 32.08 BCM/year to address the environmental flow allocation for GW recharge adequately. So, considering local water availability, the total amount of renewable water for all uses is 27.3 BCM [labeled as “New Hypothetical Sustainable System” in Fig. 6].
4 Finally, GW drawdown causes storage loss in a short-term/mid-term/long-term period. This problem is thoroughly studied and well-documented [15,16,[47], [48], [49], [50]]. Iran has already lost lots of storage capacity in GW due to subsidence, which is irreversible. It is hard to guess how much the new value for storage capacity in ground waters in Iran is now. If GW recovery shows impacts instantly and environmental flow allocation to recharge to save GWs happen right now, Iran can keep the sustainable allocation level for blue water at 27.3 BCM.

Mirzaie-Nodoushan et al. [37] presented a pareto of internal blue WF for different diets. This pareto showed 48.9 to 58.6 BCM/yr values. This estimation just for internal blue WF is possibly correct since the area for 2020 equals 148433.8 km^2^ with AET equals 55.27 BCM, which includes both blue and green water consumption. The data extracted from Iran's Ministry of agriculture shows during 2005–2014 the total croplands (both for agriculture and horticulture) was 128039.6–160347.2 km^2^. The average value for these lands was 147557.0 km^2^. It shows the area for 2020 can be a good estimation of the long-term magnitude of agriculture area in Iran.

In another study, Karandish et al. [28] estimated the internal blue WF value as 45.5 BCM for 27 major crops in Iran. Karandish et al. [28] mentioned that 78% of this consumption (35.5 BCM) is unsustainable. The 9.77 BCM difference between our estimation for 2020 and their estimation (55.27–45.5 BCM) can provide a reasonable estimate of AET from rain-fed agriculture in Iran (green internal WF). This rain-fed water consumption is out of human control, and with efficient land management, it can be a sustainable source of agricultural production. Based on [28], the maximum sustainable blue water consumption is 10 BCM/yr (45.5–35.5 BCM). Therefore, it means the maximum total sustainable water consumption in the agriculture sector in Iran (blue plus green internal WF) is around 19.77 BCM.

Karandish et al. [28] addressed that the remaining internal WF consumption is used inefficiently. MoE estimates for maximum total water consumption in agriculture show 32.8 BCM (27.3 BCM considering GW loss). Since Karandish et al. [28] provides net water consumption and MoE provides water withdrawal, their division is another reasonable estimate for the current water efficiency, which is 60% (72% considering GW loss). For efficient agriculture with a cap on water withdrawal, water efficiency should be improved to more than 60–72% levels. At the same time, Mirzaie-Nodoushan et al. [37] mentioned 106.1 BCM/yr for the total internal WF, which includes a complete diet and possibly other water usages. Our results for the maximum amount of water that agriculture can consume show 77.4 BCM from 248804.6 km^2^ of possible arable lands. This area is the maximum possible land to be used for farming/agriculture in irrigated and rain-fed systems (with SI>0). The difference between our maximum estimation (77.4 BCM) and their total internal WF (106.1 BCM) can show the extent of water use by sectors not related to both “agriculture and natural reserves.” It means around 28.7 BCM/yr is an estimate of direct evaporation without being used in any sector (either covered area like forests or areas without any coverage like deserts and mountains) and net water use by other sectors like industry and domestic. Since the reference potential evaporation in Iran is relatively high, this value in not surprising [51].

**Fig. 8:**
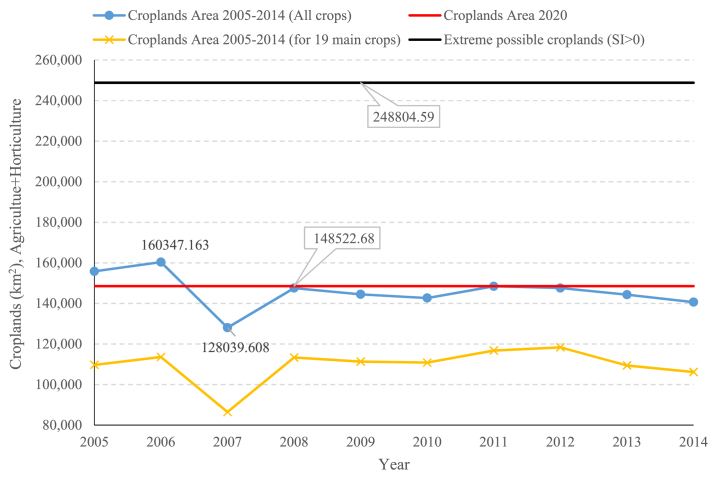
Croplands area (sum of agriculture and horticulture) time series in Iran for 2005–2014 period (km^2^).

Fig. 8 shows a maximum of 160347.16 km^2^ for total agricultural lands in 2006 and a minimum of 128039.61 for 2007 (Reference: Iran's Ministry of Agriculture Jihad). The same pattern is visible for the 19 main crops in this study. Moshir Panahi et al. [52] discussed the precipitation, water storage, and aridity ratio for 30 years in Iran, including 2005–2014. They showed Iran was in a wet condition for 2006 and dry condition for 2007. At the same time, they showed that for 2005–2014, Iran was in a “Losing Storage” state, which is visible in both SW and GW storage.

## 5 Conclusions

Iran has reached and overpassed its water-related limits to agricultural growth. To change the current water bankruptcy, policymakers in Iran should consider water-related limits to agricultural expansion. There are two sets of possible solutions to address an environmental impact, and just one of them is sustainable with guaranteed results in the long term. The first possible set is based on improving efficiency. An increase in technology application and improving efficiency cause a rebound effect and exacerbate bankruptcy and environmental problems in the long term. The second set is based on cap implementation. It means controlling water bankruptcy (as an environmental problem) should be addressed using “*heavily taxing resources or rationing them on a country basis – are thus called ‘direct’ or ‘left-side’ strategies*.” As a result, putting a cap on water use is necessary, and heavily taxing any water use beyond it is necessary to save the environment of Iran for current and future generations. This cap on net water use based on local governance is 27.3 BCM. By using this cap, society needs to find new equilibriums using new lifestyle and technology changes towards more sufficiency and efficiency. Only by seriously implementing this approach can Iranian consumers and producers work to retain the most significant welfare within the water limits given by Iran's nature.

## Author contribution

Mostafa Khorsandi: Conceived and designed the experiments; Performed the experiments; Analyzed and interpreted the data; Contributed reagents, materials, analysis tools or data; Wrote the paper. Tayebeh Omidi: Performed the experiments; Analyzed and interpreted the data; Contributed reagents, materials, analysis tools or data. Pieter Van Oel: Analyzed and interpreted the data; Contributed reagents, materials, analysis tools or data.

## Data availability statement

Data included in article/supp. material/referenced in article.

## Declaration of competing interest

The authors declare that they have no known competing financial interests or personal relationships that could have appeared to influence the work reported in this paper.
